# Epithelial-to-Pericyte Transition in Cancer

**DOI:** 10.3390/cancers9070077

**Published:** 2017-07-04

**Authors:** Jianrong Lu, Anitha K. Shenoy

**Affiliations:** 1 Department of Biochemistry and Molecular Biology, College of Medicine, University of Florida, Gainesville, FL 32610-3633, USA; 2 Department of Pharmaceutics and Biomedical Sciences, California Health Sciences University, Clovis, CA 93612, USA; ashenoy@chsu.org

**Keywords:** EMT, EPT, SRF, myocardin-related transcription factors (MRTF), pericyte, resistance, vascular niche, angiocrine factors

## Abstract

During epithelial-to-mesenchymal transition (EMT), cells lose epithelial characteristics and acquire mesenchymal properties. These two processes are genetically separable and governed by distinct transcriptional programs, rendering the EMT outputs highly heterogeneous. Our recent study shows that the mesenchymal products generated by EMT often express multiple pericyte markers, associate with and stabilize blood vessels to fuel tumor growth, thus phenotypically and functionally resembling pericytes. Therefore, some EMT events represent epithelial-to-pericyte transition (EPT). The serum response factor (SRF) plays key roles in both EMT and differentiation of pericytes, and may inherently confer the pericyte attributes on EMT cancer cells. By impacting their intratumoral location and cell surface receptor expression, EPT may enable cancer cells to receive and respond to angiocrine factors produced by the vascular niche, and develop therapy resistance.

## 1. Overview of Epithelial-to-Mesenchymal Transition (EMT) in Cancer

Most human cancers are malignancies of epithelial cells. Epithelial cells are tightly connected to each other by multiple types of intercellular junctions, which restrain their mobility. However, through a reprogramming process known as epithelial-to-mesenchymal transition (EMT), epithelial cells can dissolve cell–cell adhesions, reorganize the actin cytoskeleton, and transform into spindle-shaped mesenchymal cells with enhanced migratory and invasive capabilities [1,2]. During EMT, epithelial markers (e.g., the adherens junction protein E-cadherin and the tight junction proteins claudins) are downregulated, while mesenchymal markers (e.g., the adhesion protein N-cadherin, the intermediate filament protein vimentin, fibroblast-specific protein 1 [FSP1], and smooth muscle α-actin [SMA]) are upregulated [1,2]. The EMT program is executed in response to EMT-inducing signals that activate the expression of core transcription factors called EMT-TFs, such as Snai1/2, Zeb1/2, and Twist [1,3]. The EMT-TFs play a central role in driving EMT by directly or indirectly repressing epithelial genes.

The cardinal features of EMT have led to the prevailing hypothesis that EMT is crucial for cancer metastasis [4]. EMT liberates epithelial tumor cells from the surrounding tissue and thus promotes tumor invasion and metastatic spread. In addition, the EMT-TFs possess intrinsic activities to overcome apoptosis and oncogene-induced senescence; therefore, EMT may also foster tumor initiation [5]. Finally, EMT confers stemness-related properties and resistance to conventional radiation and chemotherapy, molecularly targeted therapy, and immunotherapy [4,6,7]. Overall, EMT exhibits multifaceted functions in tumor formation, disease progression, and therapy resistance.

However, the impact of EMT in cancer is still far from fully understood. In fact, recent studies have cast doubts on the actual contribution of EMT to metastasis [8]. In mouse models of breast cancer, lineage-tracing experiments showed that most metastatic cancer cells never activate the promoters of FSP1 and vimentin, two bona fide mesenchymal markers, and moreover, inhibition of EMT does not affect spontaneous lung metastasis formation, suggesting that EMT is dispensable for metastasis [9]. Genetic studies in mouse pancreatic cancer models demonstrated that metastasis development is independent of Snai1 and Twist [10], but partially requires Zeb1 [11]. Therefore, the functional consequences of EMT are highly context-dependent. Given the wide spectrum of intermediate phases of EMT [6], the significance of EMT in cancer remains to be elucidated. The fates and roles of epithelial tumor cells naturally transitioning to a mesenchymal state in vivo are largely elusive.

## 2. Epithelial-to-Pericyte Transition (EPT)

In our recent study [12], we conducted fate-mapping experiments to track cancer cells that undergo EMT in tumor xenografts in vivo. Breast epithelial cancer cells stably expressing an inducible form of Snai1 can be experimentally induced to initiate EMT. Such cells were fluorescently labeled and mixed with a larger number of regular epithelial cancer cells for tumor transplantation. In the resulting tumor xenografts, the majority of induced EMT cancer cells preferentially reside in the perivascular space and are associated with blood vessels, which is reminiscent of pericytes. Multiple mesenchymal cancer cell lines (including Hs578T triple-negative breast cancer cells), which are considered featuring a permanent EMT phenotype, also display similar vascular association in tumor xenografts.

Pericytes are specialized mesenchymal cells that coat and stabilize the endothelium of small blood vessels [13,14]. Pericytes are generally defined based on a combination of mesenchymal morphology, periendothelial location, and expression of multiple pericyte markers [13,14]. EMT cancer cells share a similar gene expression profile with pericytes, and indeed, EMT upregulates multiple pericyte markers in cancer cells in vitro [12]. Most Snai1-induced EMT cells in tumor xenografts express the NG2 proteoglycan, which is one of the most commonly referenced pericyte markers and is required for pericyte investment of vasculature [15,16,17]. Experimental induction of EMT substantially increases the vascular coverage by NG2-expessing cells [12].

Mammary epithelial tumor cells undergo spontaneous EMT in vivo, which is identified by the elongated cell morphology and/or an SMA promoter-driven mesenchymal-specific fluorescent reporter [12]. The majority of spontaneous EMT cells express pericyte markers NG2 and SMA, exhibit close vascular association, and apparently constitute a great proportion of pericytes associated with tumor vasculature [12]. Importantly, depletion of such naturally occurring EMT cancer cells in transplanted tumors strongly diminishes pericyte coverage, impairs vascular integrity, and attenuates tumor growth. The results suggest that EMT enables cancer cells to phenotypically and functionally resemble pericytes, and such cancer-derived pericytes are indispensable for vascular stabilization and sustained tumor growth [12].

During blood vessel maturation, endothelial cells (ECs) secrete platelet-derived growth factor (PDGF), which chemoattracts pericytes that express its cognate receptor, PDGFRβ. This paracrine signaling plays a central role in pericyte recruitment and vascular stabilization [13,14,18]. Once recruited to the abluminal surface of endothelium, pericytes make direct peg-and-socket contact or form adhesion plaques with ECs [19]. Expressed in both ECs and pericytes, N-cadherin establishes adherens junctions that strengthen the interaction between the two cell types [19,20,21]. PDGFRβ and N-cadherin are critically implicated in the pericyte-endothelium association.

EMT markedly activates the expression of both PDGFRβ and N-cadherin in cancer cells [12]. In cancer, PDGFRβ expression is generally restricted to stromal cells of mesenchymal origin, and is absent in epithelial tumor cells [22]. EMT virtually universally upregulates PDGFRβ expression [12,23,24,25,26,27,28]. Therefore, unlike epithelial cells, EMT cells are able to respond to EC-secreted chemoattractant PDGF and be recruited to vasculature. On the other hand, expression of N- and E-cadherins is usually mutually exclusive, with E-cadherin primarily expressed in epithelial cells and N-cadherin in mesenchymal cells and ECs [29]. Classical cadherins exhibit preferentially homophilic interactions. Epithelial cells are thus unable to form adherens junctions with ECs. Because the E-cadherin-to-N-cadherin switch is a hallmark of EMT [30], EMT cells acquire the new capability to associate with ECs through homotypic N-cadherin interactions. Our experimental results suggest that both PDGFRβ and N-cadherin are required for EMT cancer cells to associate with ECs in vitro and in vivo [12]. As the induction of PDGFRβ and N-cadherin is a prevalent feature of EMT, EMT may commonly confer key pericyte properties on epithelial cells, thereby often representing epithelial-to-pericyte transition (EPT).

**Figure 1:**
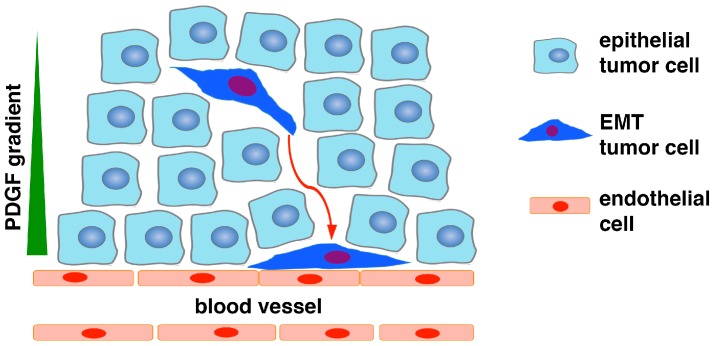
Schematic of epithelial-to-pericyte transition (EPT). In response to microenvironmental stimuli (e.g., hypoxia, transforming growth factor β TGFβ), a subset of carcinoma cells in the tumor mass undergo EMT, and consequently acquire increased motility and invasiveness as well as expression of PDGFRβ, N-cadherin, and other pericyte markers. As endothelial cells express N-cadherin and secrete PDGF, the EMT cancer cells are chemoattracted to vasculature via PDGF paracrine signaling and associate with endothelial cells through N-cadherin-mediated adherens junctions. The EMT cells may also upregulate CXCR4 and be recruited to endothelium in response to stromal-derived factor 1 (SDF1). Due to lack of PDGFRβ and N-cadherin, epithelial cancer cells without EMT are unable to respond to PDGF or interact with endothelium. The EMT cancer cells functionally resemble pericytes to stabilize blood vessels to fuel tumor growth.

Taken together, we propose that a small subset of epithelial cancer cells undergo EMT, which allows them to detach from adjoining neighboring cells and migrate within the tumor mass. Moreover, due to acquired expression of PDGFRβ and N-cadherin during EMT, the EMT cells are recruited to vasculature through PDGF-mediated chemotaxis, and subsequently establish intercellular adhesion with ECs through homodimerization of N-cadherin present on the plasma surface of both cell types (Figure 1). EMT cancer cells assume the identity of pericytes and perform pericyte function to stabilize tumor vasculature, thereby improving vascular support for the growth of the whole tumor. In short, the EPT program converts epithelial cancer cells into pericytes to fuel tumor growth.

## 3. SRF as a Potential Key Regulator of EPT

It remains largely elusive how EMT cells acquire pericyte properties. Pericyte markers are often mesenchymal markers. During EMT, while the repression of epithelial genes by EMT-TFs has been well understood, activation of the mesenchymal phenotype is much less clear. Loss of the epithelial characteristics and acquisition of the mesenchymal properties usually appear to be coupled and concomitant with each other during EMT in vitro; however, these two events are separable in vivo. For instance, transgenic expression of Snai1 in the mouse epidermis reduces E-cadherin expression and intercellular adhesion; however, mesenchymal markers are not ectopically induced in the epidermal cells [31,32]. In another example, FBXO11 is a ubiquitin ligase for Snai1/2 [33,34]. We generated FBXO11-deficient mutant mice, which showed aberrant Snai1/2 protein accumulation and transcriptionally downregulated E-cadherin expression in the epidermis, but no ectopic induction of mesenchymal markers in the mutant epidermal cells [34]. Furthermore, when lung epithelial cells are exposed to TGFβ, a prominent EMT-inducing signal, E-cadherin is downregulated and N-cadherin is upregulated, and cells undergo evident EMT. However, blocking E-cadherin downregulation does not affect N-cadherin upregulation [35]. Collectively, these observations suggest that suppression of the epithelial state and activation of the mesenchymal phenotype are independent and governed by different regulatory programs.

SMA, one of the reliable markers to characterize the mesenchymal products generated by EMT [1,2], is a well-established transcriptional target of serum response factor (SRF) [36,37,38]. SRF is a transcription factor that binds to a sequence motif known as CArG box present in many smooth muscle-specific gene promoters, and is a paramount determinant of smooth muscle differentiation [36,37,38]. Blood vessels are generally composed of two interacting cell types: ECs that form the inner lining of the vessel and mural cells that envelop the surface of the endothelial tube. Both vascular smooth muscle cells (SMCs) and pericytes are mural cells [13]. Vascular SMCs cover larger caliber blood vessels, whereas pericytes enwrap blood capillaries. The two cell types share strong phenotypic similarities [13,14]. Pericytes exhibit a number of characteristics consistent with muscle cell activity and express contractile SMA. There is no single molecular marker known that can be used to unequivocally distinguish pericytes from vascular SMCs, and “the field has generally adopted the view that pericytes belong to the same lineage and category of cells as vascular SMCs” [13,14].

SRF is widely expressed and its transcriptional activity is dependent on its coactivators. Myocardin-related transcription factors (MRTFs), including myocardin, MRTF-A, and MRTF-B, comprise a family of closely related transcriptional coactivators that physically associate with SRF and potently stimulate SRF-dependent gene expression [36,37,38]. MRTFs are regulated by actin signaling. Cytoplasmic globular actin (G-actin) retains MRTFs in the cytoplasm. Actin polymerization incorporates G-actin into filamentous actin (F-actin), thereby liberating MRTFs to enter the nucleus and interact with SRF. This activates expression of SRF-dependent genes that promote myogenic differentiation and cytoskeletal organization. Gain- and loss-of-function experiments in cultured cells and in mice have shown that MRTFs are indeed critical for vascular SMC gene activation. In addition to SMA, desmin is also a direct transcriptional target gene of SRF [39] and an established marker for pericytes and SMCs [14]. The SRF-MRTF transcriptional program is a central regulator of pericyte/vascular SMC differentiation [36,37,38]. 

Intriguingly, accumulating evidence implicates SRF and MRTFs in EMT [1]. Dynamic remodeling of actin cytoskeleton is a major event of EMT. The SRF-MRTF complex is activated by actin filament assembly [37]. The activity of SRF indeed correlates with EMT [40]. TGFβ is probably the best recognized potent inducer of EMT [1]. In renal tubular epithelial cells, TGFβ and disassembly of cell–cell junctions synergistically activate SMA expression and induce EMT [41]. MRTFs are normally localized in the cytoplasm. TGFβ triggers the nuclear translocation of MRTFs especially in epithelial cells with impaired cell–cell contacts, which subsequently act in concert with SRF to drive SMA transcription [41,42,43]. Ectopic expression of MRTFs in epithelial cells promotes EMT, whereas dominant-negative MRTF or knockdown of MRTF prevents TGFβ-induced EMT and impairs SMA induction [41,42]. In addition, the EMT-TF Zeb1 may also interact with SRF to transactivate the SMA promoter [44]. Overall, SRF and MRTFs critically activate EMT and SMA expression.

**Figure 2:**
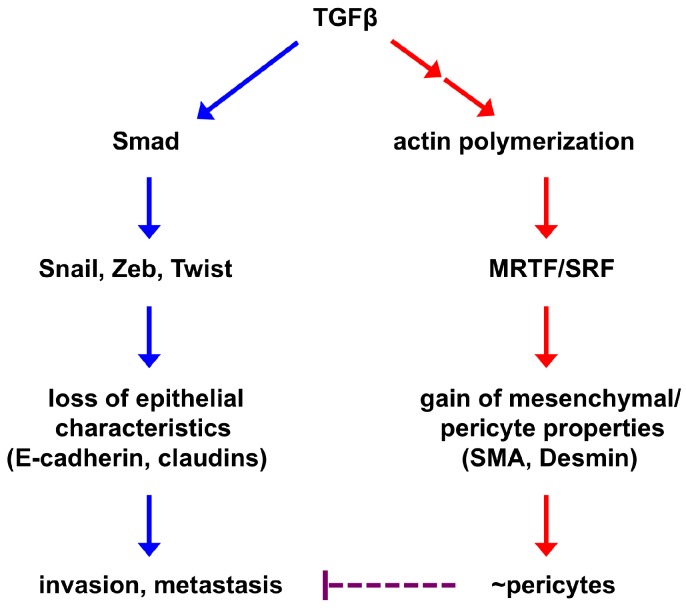
A simplified model of EPT regulation. EMT-inducing signaling causes epithelial cells to lose epithelial characteristics and acquire mesenchymal features. These two processes are largely independent of each other and governed by distinct transcriptional programs. The core EMT-TFs (Snail, Zeb, Twist) repress the epithelial phenotype and may promote tumor invasion and metastasis. Activation of the mesenchymal phenotype is at least in part mediated by the SRF transcription factor and its coactivators MRTFs, which are also central regulators of mural cells. Therefore, mesenchymal cells derived from EMT may inherently resemble pericytes and are able to associate with and stabilize blood vessels (this process is termed EPT). As EMT consists of a broad spectrum of intermediate phases, EPT is one of the EMT outputs. Improved pericyte coverage has been suggested to suppress tumor metastasis; therefore, EPT cancer cell-stabilized vasculature may impede metastatic spread of other cancer cells.

**Figure 3:**
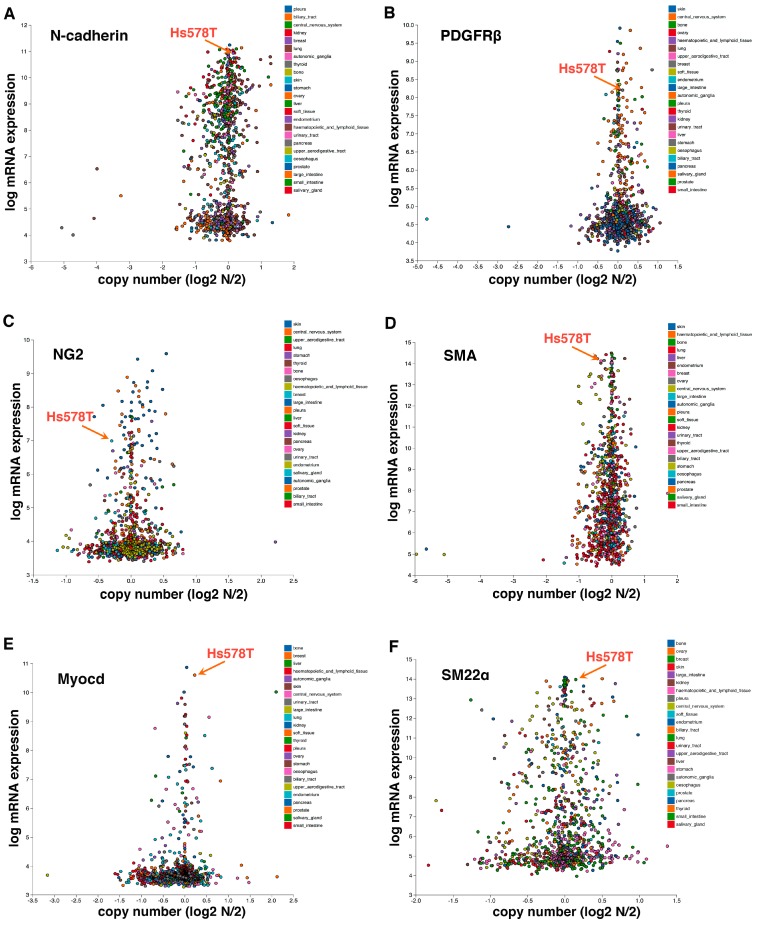
Hs578T triple-negative breast cancer cells, which behave like pericytes [12], express high levels of mesenchymal/mural cell markers N-cadherin (**A**), PDGFRβ (**B**), NG2 (**C**), SMA (**D**), Myocardin (Myocd) (**E**), and SM22α (**F**). Hs578T cells also highly express pericyte markers RGS5 and Angiopoietin 1 (Angpt.1) (data not shown). Gene expression is based on the Cancer Cell Line Encyclopedia (CCLE) database. Each dot represents an established human cancer cell line.

We postulate the following model underlying EPT regulation (Figure 2). TGFβ induces the expression of multiple EMT-TFs (primarily through the Smad signaling transducers), which in turn repress epithelial gene expression, causing the loss of epithelial features and enhancing tumor invasion and metastatic dissemination. In parallel, TGF-β signaling promotes the assembly of actin filaments from monomeric G-actin, thereby enabling nuclear import of MRTFs and their subsequent association with SRF in the nucleus to activate mesenchymal/pericyte genes. Mesenchymal acquisition is not required for metastasis [9]. Instead, because it is governed by the SRF-MRTF axis that is crucial for pericyte/SMC differentiation, mesenchymal cells resulting from EMT may inherently acquire myogenic attributes of mural cells and function like pericytes to stabilize blood vessels. Therefore, the SRF-MRTF axis may represent an intrinsic link between EMT cells and pericytes, thus supporting our discovery of EPT. Consistent with this idea, Hs578T triple-negative breast cancer cells, which behave like pericytes in our study [12], highly express multiple mesenchymal/pericyte/SMC markers, such as N-cadherin, PDGFRβ, NG2, SMA, myocardin, and smooth muscle protein 22α (SM22α or transgelin; also an established target of SRF) (Figure 3).

## 4. EPT in Development and Cancer

Pericytes are heterogeneous. Normal pericytes from different tissues may display varying morphologies, express different markers, and have diverse developmental origins [13,14,18]. Nevertheless, EMT plays a critical role in pericyte development. During embryogenesis, pericytes of the head, thymus, and outflow tract of the aorta are mostly derivatives of neural crest cells of the neuroectoderm [14,45], which is a classical model of EMT [2,4]. Pericytes in the internal viscera (such as gut, liver, lung) originate from the mesothelium that undergoes EMT [14,46,47,48]. A recent study showed that a subset of cardiac mural cells are derived from endocardial ECs through endothelial-to-mesenchymal transition [49]. Based on their outcomes, such EMT or EMT-like events during embryonic development are essentially EPT.

Pericytes in tumor vasculature may have malignant origins. It was previously observed that certain malignant melanoma and glioma cells occupy the perivascular location and interact with the abluminal surface of blood vessels “without any evidence of intravasation” [50]. While such cancer cells were proposed to migrate along the vascular surface to spread to distant sites (i.e., extravascular metastasis) [51], their vascular association is reminiscent of pericytes. Melanoma is derived from melanocyte transformation and tends to reactivate the EMT program that has enabled their neural crest ancestors to migrate during embryonic development. Malignant gliomas are often mesenchymal [52]. It is plausible that these vascular-associating melanoma and glioma cells resemble post-EMT cells. More recently, multipotent glioma stem cells (GSCs) were shown to be able to transdifferentiate into pericytes/SMCs [53,54]. Such GSC-derived cells are recruited to ECs through stromal-derived factor 1 (SDF1)-CXCR4 signaling [53], which is involved in pericyte recruitment [55]. Given that the GSC differentiation process is induced by TGFβ [53], it may activate the EMT program as well. In our study, we detected EPT in a HER2 breast carcinoma [12]. Currently it is unknown how frequently spontaneous EMT occurs in human cancer. Since claudin-low and metaplastic breast cancer subtypes are enriched for malignant mesenchymal cells and show the EMT core signature [56,57], EPT may occur in these tumors. A subset of perivascular soft tissue tumors, including glomus tumor, myopericytoma, angioleiomyoma, and liposarcoma, exhibit pericyte marker expression and perivascular growth [58,59]. Most of these tumors are presumed to originate from pericytes. It remains to be determined whether EPT-like transforming mechanisms may also contribute to pericyte marker adoption in some tumors. Overall, EPT may significantly contribute to the development of both normal and tumor pericytes.

## 5. Prospective Significance of EPT in Cancer

### 5.1. EPT in Tumor Vascularization and Growth

Judah Folkman proposed that all tumors are dependent on angiogenesis, the formation of new blood vessels [60]. Tumor growth requires vascular support. Avascular tumors are severely restricted in their growth due to the lack of a stable blood supply. Cancer cells are often able to stimulate angiogenesis for expansion of tumor mass. Nascent vessels consisting of only ECs are unstable and ineffective. Pericyte coverage is critical for vascular maturation and stability. Tumor vasculature is commonly portrayed as poorly organized, constantly remodeling, and lacking appreciable pericyte coverage [61]. However, microscopic studies have revealed the nearly ubiquitous presence of pericytes on tumor vessels, although such pericytes are typically less abundant and more loosely attached to the vasculature in tumors than in normal tissues [62]. Nevertheless, the existence of vessel-associated pericytes is vital to tumors, as experimental evidence indicates that pericytes critically maintain the integrity and functionality of the tumor vasculature. Pharmacological blockade of pericyte recruitment or genetic ablation of host-derived pericytes reduces pericyte coverage, destabilizes blood vessels, and decreases tumor growth [13,63,64]. Moreover, knockout of NG2 in mice causes pericyte deficiency and poor vessel functionality in transplanted tumors, leading to reduced tumor expansion [17]. Finally, in tumor xenografts derived from cancer cells prone to undergo EMT, a substantial fraction of pericytes are post-EMT cancer cells, and depletion of such EMT cells impairs pericyte coverage and vessel integrity, leading to diminished tumor growth [12]. The result suggests that EPT critically contributes to tumor vascularization and growth.

Severely deficient pericyte coverage destabilizes the vasculature, increases interstitial fluid pressure, and enables cancer cells to transit into the circulatory system, thus facilitating metastatic dissemination. Pericyte coverage affects breast cancer metastasis [65]. Accordingly, improved pericyte coverage may suppress tumor intravasation and metastasis [63,66]. As EPT cancer cells function like pericytes to stabilize blood vessels, they may prevent other cancer cells from intravasation and hence inhibit blood-borne metastasis (Figure 2).

### 5.2. EPT in Resistance to Anti-Angiogenesis Therapy

Vascular endothelial growth factor (VEGF) is perhaps the most important cytokine involved in tumor angiogenesis [67]. VEGF supports EC proliferation and survival. Vasculature lacking adequate pericyte coverage is vulnerable to VEGF inhibition. Anti-angiogenic therapies targeting VEGF reduce tumor vascularity and show therapeutic efficacy in human cancers, although the clinical benefits are modest and short-lived [63]. Pericytes are critical cell constituents of the tumor vasculature. Tumor pericytes express appreciable levels of VEGF and other trophic factors. Through direct support and/or paracrine interactions with ECs, pericytes mediate EC survival and protect ECs from VEGF blockade [63,68]. Tumor vessels with better pericyte coverage are less sensitive to anti-angiogenic treatment. Indeed, pericyte coverage accounts for the relative resistance of more mature vessels to VEGF withdrawal. As EPT increases pericyte coverage of the tumor vasculature, it may promote resistance to anti-angiogenic agents that target VEGF.

### 5.3. EPT in Resistance to Chemotherapy and Targeted Therapy

Conventional chemotherapy remains the backbone of treatment for most cancer patients, however, the effect is generally not long-lasting due to the emergence of drug resistance and tumor relapse. One major form of chemoresistance is attributed to EMT [7]. In tumor samples, population of residual cancer cells that survive after chemotherapy bear a gene signature with hallmarks of EMT [69,70]. Even in studies that EMT is dispensable for metastasis, the importance of EMT in chemoresistance is validated in mouse tumor models in vivo [9,10].

Activation of oncogenic pathways induces pro-growth and -survival signals on which tumors depend. This dependency of cancer cells on oncogenes, known as “oncogenic addiction”, has been exploited in the development of targeted therapy drugs. One common means by which cancer cells resist molecularly targeted therapies involves their ability to switch to a new cell type that no longer relies on the oncogenic signaling pathway being targeted by the treatments. EMT represents such a phenotypic shift in cell state that allows cancer cells to bypass pathways targeted by therapy and survive therapeutic insult [4]. Non-small-cell lung carcinomas with activating mutations in epidermal growth factor receptor (EGFR) frequently respond to treatment with tyrosine kinase inhibitors targeting EGFR, but the responses are not durable, as tumors acquire resistance. An EMT event that switches EGFR to AXL receptor tyrosine kinase is responsible for acquired resistance to EGFR inhibition [71,72,73].

EMT indeed dramatically rewires signaling pathways in cancer cells [23,24,25,26,27,28]. For instance, through the EMT process, mouse mammary epithelial tumor cells downregulate HER2 and EGFR, but upregulate PDGFRs, AXL, MET, CXCR4, etc. [23]. Consistent with the receptor changes, pre- and post-EMT cells exhibited differential responsiveness to mitogenic signals and therapeutic agents [23]. PDGFRβ expression levels correlate with tumor growth, drug resistance, and poor clinical outcomes [74]. AXL is aberrantly overexpressed in mesenchymal cells and in tumor cells refractory to therapy, and potently promotes cancer cell survival and resistance to both chemotherapy and targeted therapy [75,76,77,78]. Moreover, inappropriate activation of MET and CXCR4 is frequently implicated in resistance to conventional and targeted therapies and contributes to tumor relapse [79,80].

However, altering the landscape of receptors in EMT cells alone is insufficient to confer survival and therapy resistance, the availability of cognate ligands determines whether the newly acquired receptors and downstream signaling cascades are activated. It has been recognized that capillary ECs are not just passive conduits for delivering blood. They indeed form vascular niches that produce a variety of growth factors, cytokines, and extracellular matrix components, which are defined collectively as “angiocrine factors” [81]. The angiocrine factors act in a paracrine manner to activate survival signaling and protect responsive cells in their vicinity. It has been well established that vascular niches in the bone marrow provide a sanctuary for subpopulations of leukemic cells to resist chemotherapy-induced death [82,83,84]. Many EMT-acquired receptors can recognize EC-derived angiocrine factors. PDGF, HGF and SDF1 can activate PDGFRβ, MET and CXCR4, respectively. GAS6 is a major ligand for AXL and is present in plasma [85].

EPT enables cancer cells to occupy the periendothelial compartments, associate with blood vessels, and express cognate receptors for angiocrine factors. Therefore, EPT cancer cells are primed to respond to pro-survival signals from blood vessels and withstand the cytotoxic effects from the treatment. EPT cancer cells may have acquired stemness-like attributes during EMT [6], which may further be sustained by EC-derived angiocrine signals in the vascular niche. By contrast, non-EPT cells do not share the same receptor repertoire and/or proximity to blood vessels, and thus fail to receive the protection by vascular niches. Through the functional interactions with vascular ECs, EPT cancer cells may gain a selective survival advantage to resist chemotherapy and targeted therapy [86].

## 6. Conclusions

Through the EMT reprogramming process, epithelial cells shed epithelial characteristics and/or acquire mesenchymal properties. The two events may occur independently and each to varying extents. Therefore, EMT consists of a broad spectrum of intermediate phenotypes between the completely epithelial state and the completely mesenchymal state. The outputs of EMT are heterogeneous. Cancer cells undergoing partial EMT may acquire enhanced metastatic potential, whereas cancer cells with full EMT (in particular acquiring an SRF-driven mesenchymal phenotype) may instead resemble pericytes to stabilize tumor vasculature. Such EPT cells may be protected by the vascular niche, thus gaining increased therapy resistance and contributing to tumor relapse.

## References

[1] Lamouille S., Xu J., Derynck R. (2014). Molecular mechanisms of epithelial-mesenchymal transition. Nat. Rev. Mol. Cell Biol..

[2] Kalluri R., Weinberg R.A. (2009). The basics of epithelial-mesenchymal transition. J. Clin. Investig..

[3] Peinado H., Olmeda D., Cano A. (2007). Snail, Zeb and bHLH factors in tumour progression: An alliance against the epithelial phenotype?. Nat. Rev. Cancer.

[4] Thiery J.P., Acloque H., Huang R.Y., Nieto M.A. (2009). Epithelial-mesenchymal transitions in development and disease. Cell.

[5] Puisieux A., Brabletz T., Caramel J. (2014). Oncogenic roles of EMT-inducing transcription factors. Nat. Cell. Biol..

[6] Nieto M.A., Huang R.Y., Jackson R.A., Thiery J.P. (2016). EMT: 2016. Cell.

[7] Holohan C., Van Schaeybroeck S., Longley D.B., Johnston P.G. (2013). Cancer drug resistance: An evolving paradigm. Nat. Rev. Cancer.

[8] Diepenbruck M., Christofori G. (2016). Epithelial-mesenchymal transition (EMT) and metastasis: Yes, no, maybe?. Curr. Opin. Cell Biol..

[9] Fischer K.R., Durrans A., Lee S., Sheng J.T., Li F.H., Wong S.T.C., Choi H., Rayes T.E., Ryu S., Troeger J. (2015). Epithelial-to-mesenchymal transition is not required for lung metastasis but contributes to chemoresistance. Nature.

[10] Zheng X., Carstens J.L., Kim J., Scheible M., Kaye J., Sugimoto H., Wu C.C., LeBleu V.S., Kalluri R. (2015). Epithelial-to-mesenchymal transition is dispensable for metastasis but induces chemoresistance in pancreatic cancer. Nature.

[11] Krebs A.M., Mitschke J., Losada M.L., Schmalhofer O., Boerries M., Busch H., Boettcher M., Mougiakakos D., Reichardt W., Bronsert P. (2017). The EMT-activator Zeb1 is a key factor for cell plasticity and promotes metastasis in pancreatic cancer. Nat. Cell Biol..

[12] Shenoy A.K., Jin Y., Luo H.C., Tang M., Pampo C., Shao R., Siemann D.W., Wu L.Z., Heldermon C.D., Law B.K. (2016). Epithelial-to-mesenchymal transition confers pericyte properties on cancer cells. J. Clin. Investig..

[13] Bergers G., Song S. (2005). The role of pericytes in blood-vessel formation and maintenance. Neuro Oncol..

[14] Armulik A., Genove G., Betsholtz C. (2011). Pericytes: Developmental, physiological, and pathological perspectives, problems, and promises. Dev. Cell..

[15] Ozerdem U., Grako K.A., Dahlin-Huppe K., Monosov E., Stallcup W.B. (2001). NG2 proteoglycan is expressed exclusively by mural cells during vascular morphogenesis. Dev. Dyn..

[16] Ozerdem U., Stallcup W.B. (2004). Pathological angiogenesis is reduced by targeting pericytes via the NG2 proteoglycan. Angiogenesis.

[17] Huang F.J., Youa W.K., Bonaldob P., Seyfriedc T.N., Pasqualea E.B., Stallcupa W.B. (2010). Pericyte deficiencies lead to aberrant tumor vascularizaton in the brain of the NG2 null mouse. Dev. Biol..

[18] Diaz-Flores L., Gutierrez R., Madrid J.F., Varela H., Valladares F., Acosta E., Diaz-Flores L. (2009). Pericytes. Morphofunction, interactions and pathology in a quiescent and activated mesenchymal cell niche. Histol. Histopathol..

[19] Winkler E.A., Bell R.D., Zlokovic B.V. (2011). Central nervous system pericytes in health and disease. Nat. Neurosci..

[20] Li F., Yu L., Wang Y.L., Wang J., Yang G., Meng F.W., Han H., Meng A., Wang Y.P., Yang X. (2011). Endothelial Smad4 maintains cerebrovascular integrity by activating N-cadherin through cooperation with Notch. Dev. Cell.

[21] Gerhardt H., Wolburg H., Redies C. (2000). N-cadherin mediates pericytic-endothelial interaction during brain angiogenesis in the chicken. Dev. Dyn..

[22] Heldin C.H., Westermark B. (1999). Mechanism of action and in vivo role of platelet-derived growth factor. Physiol. Rev..

[23] Jahn S.C., Law M.E., Corsino P.E., Parker N.N., Pham K., Davis B.J., Lu J.R., Law B.K. (2012). An in vivo model of epithelial to mesenchymal transition reveals a mitogenic switch. Cancer Lett..

[24] Campbell C.I., Moorehead R.A. (2011). Mammary tumors that become independent of the type I insulin-like growth factor receptor express elevated levels of platelet-derived growth factor receptors. BMC Cancer.

[25] Jechlinger M., Grunert S., Tamir I.H., Janda E., Ludemann S., Waerner T., Seither P., Weith A., Beug H., Kraut N. (2003). Expression profiling of epithelial plasticity in tumor progression. Oncogene.

[26] Jechlinger M., Sommer A., Moriggl R., Seither P., Kraut N., Capodiecci P., Donovan M., Cordon-Cardo C., Beug H., Grünert S. (2006). Autocrine PDGFR signaling promotes mammary cancer metastasis. J. Clin. Investig..

[27] Steller E.J., Raats D.A., Koster J., Rutten B., Govaert K.M., Emmink B.L., Snoeren N., van Hooff S.R., Holstege F.C., Maas C. (2013). PDGFRB promotes liver metastasis formation of mesenchymal-like colorectal tumor cells. Neoplasia.

[28] Thomson S., Petti F., Sujka-Kwok I., Epstein D., Haley J.D. (2008). Kinase switching in mesenchymal-like non-small cell lung cancer lines contributes to EGFR inhibitor resistance through pathway redundancy. Clin. Exp. Metastasis.

[29] Van Roy F. (2014). Beyond E-cadherin: Roles of other cadherin superfamily members in cancer. Nat. Rev. Cancer.

[30] Wheelock M.J., Shintani Y., Maeda M., Fukumoto Y., Johnson K.R. (2008). Cadherin switching. J. Cell Sci..

[31] Jamora C., Lee P., Kocieniewski P., Azhar M., Hosokawa R., Chai Y., Fuchs E. (2005). A signaling pathway involving TGF-beta2 and snail in hair follicle morphogenesis. PLoS Biol..

[32] Du F., Nakamura Y., Tan T.L., Lee P., Lee R., Yu B., Jamoraet C. (2010). Expression of snail in epidermal keratinocytes promotes cutaneous inflammation and hyperplasia conducive to tumor formation. Cancer Res..

[33] Zheng H., Shen M., Zha Y.L., Li W.Y., Wei Y., Blanco M.A., Ren G.W., Zhou T.H., Storz P., Wang H.Y. (2014). PKD1 phosphorylation-dependent degradation of SNAIL by SCF-FBXO11 regulates epithelial-mesenchymal transition and metastasis. Cancer Cell.

[34] Jin Y., Shenoy A.K., Doernberg S., Chen H., Luo H.C., Shen H.X., Lin T., Tarrash M., Cai Q.S., Hu X. (2015). FBXO11 promotes ubiquitination of the Snail family of transcription factors in cancer progression and epidermal development. Cancer Lett..

[35] Tang M., Shen H., Jin Y., Lin T., Cai Q., Pinard M.A., Biswas S., Tran Q., Li G., Shenoy A.K. (2013). The malignant brain tumor (MBT) domain protein SFMBT1 is an integral histone reader subunit of the LSD1 demethylase complex for chromatin association and epithelial-to-mesenchymal transition. J. Biol. Chem..

[36] Wang D.Z., Olson E.N. (2004). Control of smooth muscle development by the myocardin family of transcriptional coactivators. Curr. Opin. Genet. Dev..

[37] Olson E.N., Nordheim A. (2010). Linking actin dynamics and gene transcription to drive cellular motile functions. Nat. Rev. Mol. Cell Biol..

[38] Parmacek M.S. (2007). Myocardin-related transcription factors: Critical coactivators regulating cardiovascular development and adaptation. Circ. Res..

[39] Mericskay M., Parlakian A., Porteu A., Dandre F., Bonnet J., Paulin D., Li Z.L. (2000). An overlapping CArG/octamer element is required for regulation of desmin gene transcription in arterial smooth muscle cells. Dev. Biol..

[40] Psichari E., Balmain A., Plows D., Zoumpourlis V., Pintzas A. (2002). High activity of serum response factor in the mesenchymal transition of epithelial tumor cells is regulated by RhoA signaling. J. Biol. Chem..

[41] Fan L., Sebe A., Peterfi Z., Masszi A., Thirone A.C., Rotstein O.D., Nakano H., McCulloch C.A., Szaszi K., Mucsi I. (2007). Cell contact-dependent regulation of epithelial-myofibroblast transition via the rho-rho kinase-phospho-myosin pathway. Mol. Biol. Cell.

[42] Morita T., Mayanagi T., Sobue K. (2007). Dual roles of myocardin-related transcription factors in epithelial mesenchymal transition via slug induction and actin remodeling. J. Cell Biol..

[43] Busche S., Descot A., Julien S., Genth H., Posern G. (2008). Epithelial cell-cell contacts regulate SRF-mediated transcription via Rac-actin-MAL signalling. J. Cell Sci..

[44] Nishimura G., Manabe I., Tsushima K., Fujiu K., Oishi Y., Imai Y., Maemura K., Miyagishi M., Higashi Y., Kondoh H. (2006). DeltaEF1 mediates TGF-beta signaling in vascular smooth muscle cell differentiation. Dev. Cell..

[45] Trost A., Schroedl F., Lange S., Rivera F.J., Tempfer H., Korntner S., Stolt C.C., Wegner M., Bogner B., Kaser-Eichberger A. (2013). Neural crest origin of retinal and choroidal pericytes. Invest. Ophthalmol. Vis. Sci..

[46] Wilm B., Ipenberg A., Hastie N.D., Burch J.B., Bader D.M. (2005). The serosal mesothelium is a major source of smooth muscle cells of the gut vasculature. Development.

[47] Que J., Wilm B., Hasegawa H., Wang F., Bader D., Hogan B.L.M. (2008). Mesothelium contributes to vascular smooth muscle and mesenchyme during lung development. Proc. Natl. Acad. Sci. USA.

[48] Asahina K., Zhou B., Pu W.T., Tsukamoto H. (2011). Septum transversum-derived mesothelium gives rise to hepatic stellate cells and perivascular mesenchymal cells in developing mouse liver. Hepatology.

[49] Chen Q., Zhang H., Liu Y., Adams S., Eilken H., Stehling M., Corada M., Dejana E., Zhou B., Adams R.H. (2016). Endothelial cells are progenitors of cardiac pericytes and vascular smooth muscle cells. Nat. Commun..

[50] Lugassy C., Haroun R.I., Brem H., Tyler B.M., Jones R.V., Fernandez P.M., Patierno S.R., Kleinman H.K., Barnhill R.L. (2002). Pericytic-like angiotropism of glioma and melanoma cells. Am. J. Dermatopathol..

[51] Lugassy C., Peault B., Wadehra M., Kleinman H.K., Barnhill R.L. (2013). Could pericytic mimicry represent another type of melanoma cell plasticity with embryonic properties?. Pigment. Cell Melanoma. Res..

[52] Olar A., Aldape K.D. (2014). Using the molecular classification of glioblastoma to inform personalized treatment. J. Pathol..

[53] Cheng L., Huang Z., Zhou W., Wu Q., Donnola S., Liu J.K., Fang X., Sloan A.E., Mao Y., Lathia J.D. (2013). Glioblastoma stem cells generate vascular pericytes to support vessel function and tumor growth. Cell.

[54] Shao R., Taylor S.L., Oh D.S., Schwartz L.M. (2015). Vascular heterogeneity and targeting: The role of YKL-40 in glioblastoma vascularization. Oncotarget.

[55] Song N., Huang Y., Shi H., Yuan S., Ding Y., Song X., Fu Y., Luo Y. (2009). Overexpression of platelet-derived growth factor-BB increases tumor pericyte content via stromal-derived factor-1alpha/CXCR4 axis. Cancer Res..

[56] Taube J.H., Herschkowitz J.I., Komurov K., Zhou A.Y., Gupta S., Yang J., Hartwell K., Onder T.T., Gupta P.B., Evans K.W. (2010). Core epithelial-to-mesenchymal transition interactome gene-expression signature is associated with claudin-low and metaplastic breast cancer subtypes. Proc. Natl. Acad. Sci. USA.

[57] Prat A., Perou C.M. (2011). Deconstructing the molecular portraits of breast cancer. Mol. Oncol..

[58] Shen J., Shrestha S., Rao P.N., Asatrian G., Scott M.A., Nguyen V., Giacomelli P., Soo C., Ting K., Eilber F.C. (2016). Pericytic mimicry in well-differentiated liposarcoma/atypical lipomatous tumor. Hum. Pathol..

[59] Shen J., Shrestha S., Yen Y.H., Asatrian G., Mravic M., Soo C., Ting K., Dry S.M., Peault B., James A.W. (2015). Pericyte Antigens in Perivascular Soft Tissue Tumors. Int. J. Surg. Pathol..

[60] Folkman J. (2006). Angiogenesis. Annu. Rev. Med..

[61] Carmeliet P., Jain R.K. (2011). Molecular mechanisms and clinical applications of angiogenesis. Nature.

[62] Morikawa S., Baluk P., Kaidoh T., Haskell A., Jain R.K., McDonald D.M. (2002). Abnormalities in pericytes on blood vessels and endothelial sprouts in tumors. Am. J. Pathol..

[63] Bergers G., Hanahan D. (2008). Modes of resistance to anti-angiogenic therapy. Nat. Rev. Cancer.

[64] Cooke V.G., LeBleu V.S., Keskin D., Khan Z., O’Connell J.T., Teng Y., Duncan M.B., Xie L., Maeda G., Vong S. (2012). Pericyte depletion results in hypoxia-associated epithelial-to-mesenchymal transition and metastasis mediated by met signaling pathway. Cancer Cell.

[65] Kim J., de Sampaio P.C., Lundy D.M., Peng Q., Evans K.W., Sugimoto H., Gagea M., Kienast Y., do Amaral N.S., Rocha R.M. (2016). Heterogeneous perivascular cell coverage affects breast cancer metastasis and response to chemotherapy. JCI Insight.

[66] Gerhardt H., Semb H. (2008). Pericytes: Gatekeepers in tumour cell metastasis?. J. Mol. Med. (Berl.).

[67] Ferrara N., Adamis A.P. (2016). Ten years of anti-vascular endothelial growth factor therapy. Nat. Rev. Drug. Discov..

[68] Van Beijnum J.R., Nowak-Sliwinska P., Huijbers E.J., Thijssen V.L., Griffioen A.W. (2015). The great escape; the hallmarks of resistance to antiangiogenic therapy. Pharmacol. Rev..

[69] Dave B., Mittal V., Tan N.M., Chang J.C. (2012). Epithelial-mesenchymal transition, cancer stem cells and treatment resistance. Breast Cancer Res..

[70] Creighton C.J., Li X., Landis M., Dixon J.M., Neumeister V.M., Sjolund A., Rimm D.L., Wong H., Rodriguez A., Herschkowitz J.I. (2009). Residual breast cancers after conventional therapy display mesenchymal as well as tumor-initiating features. Proc. Natl. Acad. Sci. USA.

[71] Zhang Z., Lee J.C., Lin L., Olivas V., Au V., LaFramboise T., Abdel-Rahman M., Wang X., Levine A.D., Rho J.K. (2012). Activation of the AXL kinase causes resistance to EGFR-targeted therapy in lung cancer. Nat. Genet..

[72] Byers L.A., Diao L., Wang J., Saintigny P., Girard L., Peyton M., Shen L., Fan Y., Giri U., Tumula P.K. (2013). An epithelial-mesenchymal transition gene signature predicts resistance to EGFR and PI3K inhibitors and identifies Axl as a therapeutic target for overcoming EGFR inhibitor resistance. Clin. Cancer Res..

[73] Thomson S., Buck E., Petti F., Griffin G., Brown E., Ramnarine N., Iwata K.K., Gibson N., Haley J.D. (2005). Epithelial to mesenchymal transition is a determinant of sensitivity of non-small-cell lung carcinoma cell lines and xenografts to epidermal growth factor receptor inhibition. Cancer Res..

[74] Cao Y. (2013). Multifarious functions of PDGFs and PDGFRs in tumor growth and metastasis. Trends Mol. Med..

[75] Graham D.K., DeRyckere D., Davies K.D., Earp H.S. (2014). The TAM family: Phosphatidylserine sensing receptor tyrosine kinases gone awry in cancer. Nat. Rev. Cancer.

[76] Wu X., Liu X., Koul S., Lee C.Y., Zhang Z., Halmos B. (2014). AXL kinase as a novel target for cancer therapy. Oncotarget.

[77] Scaltriti M., Elkabets M., Baselga J. (2016). Molecular Pathways: AXL, a Membrane Receptor Mediator of Resistance to Therapy. Clin. Cancer Res..

[78] Wang C., Jin H., Wang N., Fan S., Wang Y., Zhang Y., Wei L., Tao X., Gu D., Zhao F. (2016). Gas6/Axl Axis Contributes to Chemoresistance and Metastasis in Breast Cancer through Akt/GSK-3beta/beta-catenin Signaling. Theranostics.

[79] Corso S., Giordano S. (2013). Cell-autonomous and non-cell-autonomous mechanisms of HGF/MET-driven resistance to targeted therapies: From basic research to a clinical perspective. Cancer Discov..

[80] Chatterjee S., Behnam A.B., Nimmagadda S. (2014). The intricate role of CXCR4 in cancer. Adv. Cancer Res..

[81] Rafii S., Butler J.M., Ding B.S. (2016). Angiocrine functions of organ-specific endothelial cells. Nature.

[82] Butler J.M., Kobayashi H., Rafii S. (2010). Instructive role of the vascular niche in promoting tumour growth and tissue repair by angiocrine factors. Nat. Rev. Cancer.

[83] Tabe Y., Konopleva M. (2014). Advances in understanding the leukaemia microenvironment. Br. J. Haematol..

[84] Doan P.L., Chute J.P. (2012). The vascular niche: Home for normal and malignant hematopoietic stem cells. Leukemia.

[85] Laurance S., Lemarie C.A., Blostein M.D. (2012). Growth arrest-specific gene 6 (gas6) and vascular hemostasis. Adv. Nutr..

[86] Shenoy A.K., Lu J. (2017). Relevance of epithelial-to-pericyte transition in cancer. Mol. Cell. Oncol..

